# OsKEAP1 Interacts with OsABI5 and Its Downregulation Increases the Transcription of *OsABI5* and the ABA Response Genes in Germinating Rice Seeds

**DOI:** 10.3390/plants10030527

**Published:** 2021-03-11

**Authors:** Yan-Hua Liu, Meng Jiang, Rui-Qing Li, Jian-Zhong Huang, Qing-Yao Shu

**Affiliations:** 1 National Key Laboratory of Rice Biology and Zhejiang Provincial Key Laboratory of Crop Germplasm Resources, Institute of Crop Sciences, Zhejiang University, Hangzhou 310058, China; 11616004@zju.edu.cn (Y.-H.L.); mengjiang@zju.edu.cn (M.J.); jzhuang@zju.edu.cn (J.-Z.H.); 2 College of Agronomy, Anhui Agricultural University, Hefei 230036, China; 11416095@zju.edu.cn; 3 Key Laboratory for Nuclear Agricultural Sciences of Zhejiang Province and Ministry of Agriculture and Rural Affairs, Institute of Nuclear Agricultural Sciences, Zhejiang University, Hangzhou 310058, China

**Keywords:** seed germination, abscisic acid (ABA), OsKEAP1, OsABI1, OsABI2, OsABI5, Os01g0162500, Os05g0164900, *Oryza sativa* L.

## Abstract

Kelch-like ECH-associated protein 1 (KEAP1)–nuclear factor E2-related factor 2 (NRF2) is the key antioxidant system in animals. In a previous study, we identified a probable KEAP1 ortholog in rice, OsKEAP1, and demonstrated that the downregulation of *OsKEAP1* could alter the redox system and impair plant growth, as well as increase the susceptibility to abscisic acid (ABA) in seed germination. However, no NRF2 orthologs have been identified in plants and the mechanism underlying the phenotype changes of downregulated *oskeap1* mutants is yet unknown. An in silico search showed that OsABI5 is the gene that encodes a protein with the highest amino acid identity score (38.78%) to NRF2 in rice. In this study, we demonstrated that, via yeast two-hybrids analysis and bimolecular fluorescence complementation assays, OsKEAP1 interacted with OsABI5 via its Kelch repeat domain in the nucleus. In germinating seeds, the expression of *OsKEAP1* was significantly downregulated in *oskeap1-1* (39.5% that of the wild-type (WT)) and *oskeap1-2* (64.5% that of WT), while the expression of *OsABI5* was significantly increased only in *oskeap1-1* (247.4% that of WT) but not in *oskeap1-2* (104.8% that of WT). ABA (0.5 μM) treatment significantly increased the expression of *OsKEAP1* and *OsABI5* in both the *oskeap1* mutants and WT, and 4 days post treatment, the transcription level of *OsABI5* became significantly greater in *oskeap1-1* (+87.2%) and *oskeap1-2* (+55.0%) than that in the WT. The ABA-responsive genes (*OsRab16A* and three *late embryogenesis abundant* genes), which are known to be activated by OsABI5, became more responsive to ABA in both *oskeap1* mutants than in the WT. The transcript abundances of genes that regulate OsABI5, e.g., *OsSnRK2* (encodes a kinase that activates OsABI5), *OsABI1*, and *OsABI2* (both encode proteins binding to OsSnRK2 and are involved in ABA signaling) were not significantly different between the two *oskeap1* mutants and the WT. These results demonstrated that OsKEAP1 played a role in the ABA response in rice seed germination via regulating OsABI5, which is the key player in the ABA response. In-depth analyses of the components and their action mode of the KEAP1–NRF2 and ABA signaling pathways suggested that OsKEAP1 likely formed a complex with OsABI5 and OsKEG, and OsABI5 was ubiquitinated by OsKEG and subsequently degraded under physiological conditions; meanwhile, under oxidative stress or with increased an ABA level, OsABI5 was released from the complex, phosphorylated, and transactivated the ABA response genes. Therefore, OsKEAP1–OsABI5 bore some resemblance to KEAP1–NRF2 in terms of its function and working mechanism.

## 1. Introduction

Cells have developed a variety of antioxidant mechanisms that provide a defense against various redox stresses. The Kelch-like ECH-associated protein 1 (KEAP1)–nuclear factor E2-related factor (NRF2)–antioxidant response element (ARE) system has been recognized as one of the prevailing systems involved in the antioxidant response in animals (see recent reviews by [1,2,3,4,5,6]). In brief, under normal physiological conditions, NRF2 is sequestered in the cytoplasm by KEAP1 and is constantly ubiquitinated in the Keap1–NRF2–Cul3–E3 ubiquitin ligase complex, and eventually degraded via proteasome-dependent degradation. Under stressed conditions, NRF2 is dissociated from KEAP1 and translocated to the nucleus, where it is phosphorylated and heterodimerized with one of the small Maf (musculoaponeurotic fibrosarcoma oncogene homolog) proteins and ultimately binds to AREs, activating target genes with ARE in their promoters. KEAP1 can also interact with other molecules, such as p62, allowing for the accumulation of endogenous p62 or ectopic expression of p62 sequesters KEAP1 into aggregates, resulting in the inhibition of KEAP1-mediated NRF2 ubiquitination; hence, KEAP1 regulators and molecules could also indirectly regulate NRF2 [7,8,9,10].

The KEAP1–NRF2 pathway is known to be present in organisms from arthropods to mammals, but whether a similar KEAP1–NRF2 system exists in plants remains inconclusive [11]. Bioinformatics analysis has indicated that there is no close KEAP1 or NRF2 ortholog in plants [12]. Reactive oxygen species (ROS) are produced through various metabolic processes in almost all living plant cells; there are many forms ROS, e.g., singlet oxygen (^1^O_2_), hydroxyl (OH^•^) radicals, and hydrogen peroxide (H_2_O_2_). Among them, all but H_2_O_2_ have a very short half-life; hence, H_2_O_2_ is regarded as the predominant ROS involved in cellular signaling. ROS is best known for being involved in biotic and abiotic stress responses, but recent studies have revealed they are also heavily involved in plant development processes (see the review by Mhamdi and Breusegem [13]). Therefore, ROS indeed plays similar roles in the growth and defense of plants and animals; hence, we contemplate that it might be worthwhile to explore the KEAP1–NRF2 system in plants.

As a start, we identified a probable KEAP1 ortholog in rice, OsKEAP1, and investigated its function by generating two downregulated mutants, *oskeap1-1* and *oskeap1-2*, via gene editing (we failed to produce any knockout mutants), and demonstrated that the downregulation of *OsKEAP1* resulted in defective seed and plant growth and development [14]. Of particular relevance, the two mutants showed altered redox homeostasis and became more susceptible to abscisic acid (ABA) in seed germination [14].

ABA regulates various biological programs in seed development and germination, with the ABI5 as the integrator of ABA signaling (see reviews [15,16]). Briefly, when no ABA or little ABA is present, ABI1 and ABI2, which are both forms of protein phosphatase 2C (PP2C), bind to SNF1-related protein kinase 2 (SnRK2), which suppresses its kinase activity and makes it incapable of activating ABI5 via phosphorylation. When ABA is present, the ABA receptor binds to PP2C and, thus, the SnRK2 is able to phosphorylate and activate ABI5, which consequently activates the expression of its target genes with ABA response element (ABRE, an eight-base pair sequence CACGTGGC) in their promoter [16]. At the transcription level, many transcription factors, such as ABI3, ABI4, MYB7, and WRKYs, play either a positive or a negative role in the regulation of *ABI5* expression [15]. At the post-translational level, ABI5 is subjected to ubiquitination by KEEP ON GOING (KEG), a RING-type E3 ligase [17,18], and ubiquitinated ABI5 is subsequently destabilized and degraded [19].

The rice ABI5 ortholog, OsABI5, was first identified and characterized as a bZIP transcription factor by Zou et al. [20]. They soon demonstrated that OsABI5 is involved in rice fertility and stress tolerance: ABA and high salinity upregulated *OsABI5*, while drought and cold treatments downregulated *OsABI5*; furthermore, the repression of *OsABI5* promoted stress tolerance and resulted in low fertility [21]. Mutation of *Preharvesting Spouting* (*PHS*) *8* (the isoamylase 1 gene) could decrease the transcription of *OsABI5* (and *OsABI3*) [22]. Recently, Sakuraba et al. [23] demonstrated that the rice NAM/ATAF1/2/CUC2 (NAC) transcription factor ONAC054, which can activate the transcription of *OsABI5* by binding to its promoter, plays an important role in ABA-induced leaf senescence. ABI5 orthologs have also been identified in other important crops with a similar function, such as in maize (ZmABI5 [24]), *Brassica oleracea* (BoIABI5, [25]), barley (HvABI5 [26]), and legumes [27]. As reviewed by Skubacz et al. [15], ABI5 orthologs in plants are involved in the regulation of a broad range of activities from the early developmental processes, adaptation, and response to unfavorable environmental conditions.

A brief search for NRF2 homologs in the rice genome showed that OsABI5 had the highest amino acid identity score of 38.78%. NRF2 and OsABI5 consist of 605 amino acids (aa) and 388 aa, respectively, but both contain a bZIP domain at their C termini (Figure S1, Supplemental Datasheet 1). Probably due to the low homology of OsABI5 to NRF2 proteins of nonplant organisms, we failed to build a phylogenetic tree that included the NRF2 homologs of both animals and plants, and hence, OsABI5 cannot be directly considered as an NRF2 ortholog. However, the known function and functioning mode of plant ABI5 orthologs seem to be somewhat similar to those of NRF2; hence, we contemplated that OsKEAP1 possibly regulates both the redox and ABA responses via OsABI5. In the present study, we first investigated whether OsKEAP1 interacted with OsABI5 in a way that is similar to KEAP1 interacting with NRF2, and then examined the effect of *OsKEAP1* downregulation on the expression of *OsABI5* and genes that regulate or are regulated by OsABI5. We aimed to learn more about the role and mechanism of OsKEAP1 in ABA and ROS regulation in plants in general and in rice in particular, and how much the KEAP1–NRF2 system may retain in plants.

## 2. Results

### 2.1. OsKEAP1 Interacts with OsABI5 in the Nucleus

**Figure 1:**
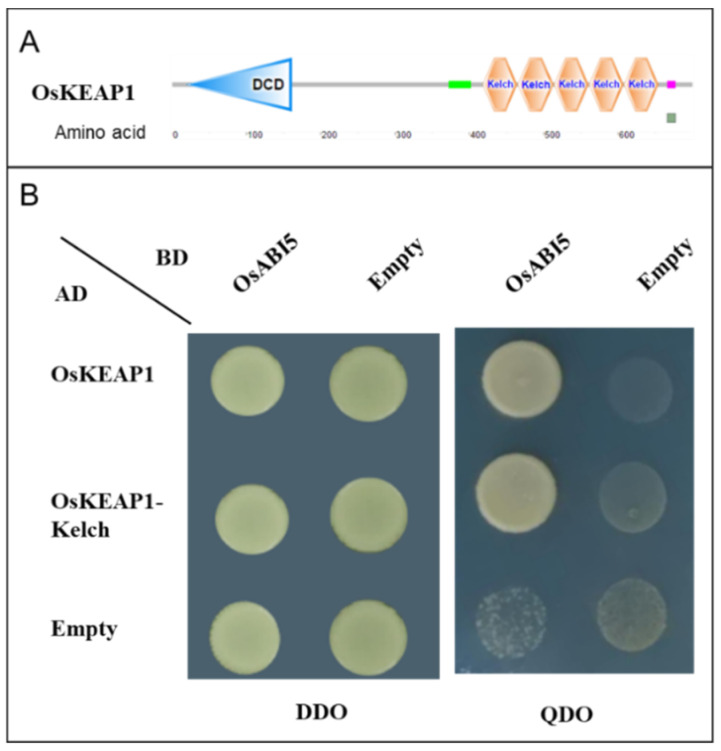
Yeast two-hybrid (Y2H) analysis of OsKEAP1 and its Kelch repeat domain (OsKelch) with OsABI5. (**A**) Diagram of OsKEAP1 showing the position of the OsKelch domain. Prediction of the domains was performed at https://smart.embl.de/ (accessed on 5 March 2021). DCD: Development and Cell Death. The green and pink/red boxes represent the coiled coil region and low-complexity region, respectively. (**B**) Y2H analysis. Yeast cells were transformed with BD-OsABI5 and AD-OsKEAP1 or AD-OsKelch, as indicated. Yeast cells were incubated on DDO (double dropout: SD/-Leu/-Trp) and QDO (quadruple dropout: SD/-Ade/-His/-Leu/-Trp) plates for 5 days.

KEAP1 is known to interact with NRF2 via its Kelch repeat domain in the cytoplasm [28] in animals. We deployed the yeast two-hybrid (Y2H) assay to assess the interaction between OsKEAP1 (Figure 1A) and OsABI5. We first demonstrated that OsKEAP1 interacted with OsABI5 (Figure 1B, upper lane), and then further confirmed that the Kelch repeat domain of OsKEAP1 alone could also interact with OsABI5 (Figure 1B, middle lane). These results demonstrated that OsKEAP1 interacts with OsABI5 in the same way that as KEAP1 does with NRF2.

Our previous study showed that OsKEAP1 is localized to both the cytoplasm and nucleus in rice protoplasts [14], unlike KEAP1, which is mainly localized in the cytoplasm [29]. Zou et al. [20] demonstrated that OsABI5 is present in the nucleus; our assay also confirmed that OsABI5 is localized to the nucleus (Figure S2).

**Figure 2:**
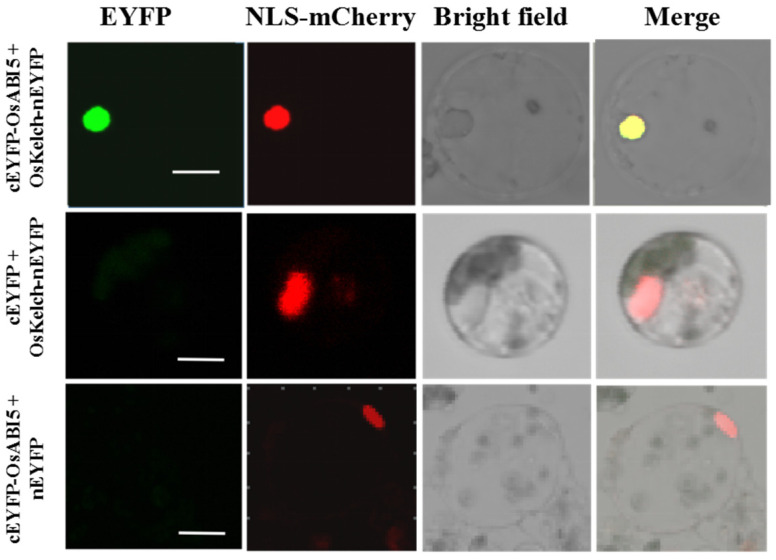
Bimolecular fluorescence complementation (BiFC) analysis of the interaction between OsABI5 and the Kelch repeat domain of OsKEAP1 (OsKelch) in rice protoplasts. NLS-mCherry: fluorescence of nuclear localization signal-mCherry; Merge: merge of the EYFP, NLS-mCherry, and brightfield images. Scale bars: 5 µm.

To investigate whether OsKEAP1 interacts with OsABI5 in the nucleus, we performed bimolecular fluorescence complementation (BiFC) assays by transiently coexpressing a series of vector combinations into rice protoplasts. Because it is the Kelch domain of OsKEAP1 that interacts with OsABI5, as demonstrated in the Y2H assay (Figure 1), we constructed vectors using the Kelch domain (OsKelch) instead of the whole OsKEAP1 sequence. Among them, the cEYFP–OsABI5/OsKelch–nEYFP combination produced enhanced yellow fluorescence protein (EYFP) in the nucleus (Figure 2), demonstrating a direct interaction between them in the nucleus of rice protoplasts.

### 2.2. Downregulation of OsKEAP1 Enhanced the Transcription of OsABI5

In mammals, KEAP1 regulates NRF2 at the post-translational level by binding to NRF2 in the cytoplasm [5,6]. To assess whether the *OsKEAP1* downregulation also affected *OsABI5* transcription, qRT-PCR was performed to detect the abundance of the transcripts of *OsABI5* in the two *oskeap1* mutants and their wild-type parent Xidao #1 (WT). Our previous results showed that, at both the seedling and heading stages, the two *oskeap1* mutants grown under normal conditions had significantly less abundant *OsKEAP1* transcripts than the WT. Furthermore, the expression of *OsKEAP1* is lower in *oskeap1-1* than in *oskeap1-2* [14].

**Figure 3:**
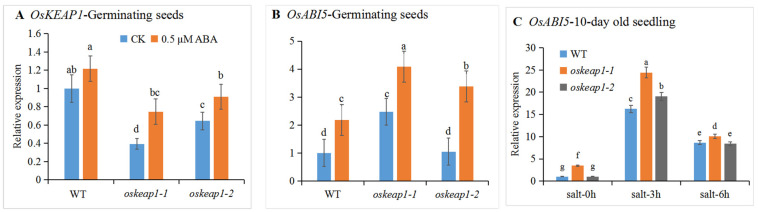
Relative expression of *OsKEAP1* and *OsABI5* in germinating seeds and seedlings. (**A**,**B**) Germinating seeds 4 days after germinating on a 0.5 MS medium without abscisic acid (ABA) (CK) or with 0.5 μM ABA. (**C**) Ten-day-old seedlings subject to 200 mM NaCl for 3 h (salt-3 h) or 6 h (salt-6 h) or without NaCl treatment (salt-0 h). The expression levels of the same gene in different materials or grown under different conditions were compared with the wild-type (WT) grown without the treatment (its level was set as 1). *OsACTIN* was used as an internal reference. Data are shown in mean ± standard error of three biological repeats. One-way ANOVA was performed for the statistical analysis, where different letters represent significant differences (*p* ≤ 0.05).

In 4-day-old germinating seeds, the expression of *OsKEAP1* in the two *oskeap1* mutants was also significantly lower compared with the WT, both with or without the ABA (0.5 μM) treatment. When germinated without the ABA treatment, *oskeap1-1* and *oskeap1-2* had *OsKEAP1* transcript abundances of 39.5% and 64.6% that of the WT, respectively (Figure 3A). When germinated with ABA, the transcription of *OsKEAP1* increased significantly in both *oskeap1-1* (89.2%) and *oskeap1-2* (41.0%), but was not significantly changed in the WT. Nevertheless, the *OsKEAP1* transcript abundances of the two mutants in the presence of exogenous ABA were still much lower than the WT, i.e., 61.4% (*oskeap1-1*) and 74.7% (*oskeap1-2*) that of the WT. These results suggest that the mutants were more responsive to ABA treatment than the WT.

When germinating in the absence of ABA treatment, the expression of *OsABI5* was significantly greater in *oskeap1-1*, with a transcript abundance that was 2.47 times that of the WT, while *oskeap1-2* showed no significant difference from the WT (Figure 3B). The 0.5 μM ABA treatment significantly increased the *OsABI5* expression in both the *oskeap1* mutants and the WT, and their transcript abundance reached 4.08 (*oskeap1-1*), 3.38 (*oskeap1-2*), and 2.18 (WT) times that of the WT without the ABA treatment (or an increase of 87.2% and 55.0% in *oskeap1-1* and *oskeap1-2*, respectively, compared with the WT with ABA treatment) (Figure 3B), confirming that *OsABI5* is responsive to ABA treatment, as previously reported. Overall, the changing trend of the expression levels of *OsABI5* in *oskeap1-1* and *oskeap1-2* as compared with the WT were consistent with those of *OsKEAP1*, particularly when treated with ABA. 

In our previous study, we reported that *oskeap1-1* and *oskeap1-2* had significantly lower *OsKEAP1* expression in leaf tissues of 10-day-old seedlings than the WT, and salt significantly increased *OsKEAP1* expression 3 and 6 h after treatment in both mutants and WT [14]. We checked the expression level of *OsABI5* in those materials and a similar trend was observed: the salt treatment significantly increased the *OsABI5* expression in all materials but its highest level was always in *oskeap1-1*. Significant differences between the WT and *oskeap1-2* were observed 3 h after the salt treatment (Figure 3C).

### 2.3. Downregulation of OsKEAP1 Had Little Effect on the Expression of Genes Regulating OsABI5

**Figure 4:**
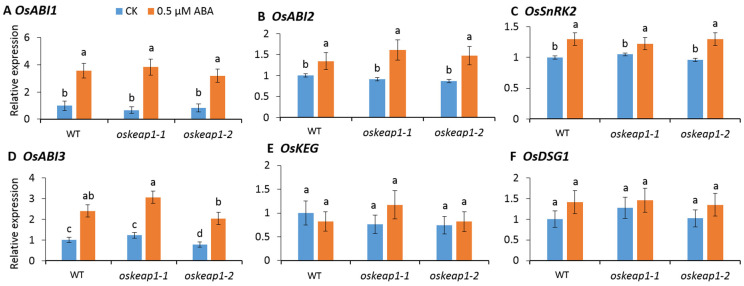
Relative expression of genes involved in the ABA signal transduction in seeds 4 days after germinating on a 0.5 MS medium without abscisic acid (ABA) (CK) or with 0.5 μM ABA. The expression levels of the same gene in different materials were compared with the wild-type (WT) germinated without ABA (its level was set as 1). *OsACTIN* was used as an internal reference. Data are shown as the mean ± standard error of three biological repeats. One-way ANOVA was performed for the statistical analysis, where different letters represent significant differences (*p* ≤ 0.05).

OsABI5 is known to be regulated at the post-translational level by various genes [16]. Hence, it is of interest to examine whether the downregulation of *OsKEAP1* might have an effect on the transcription of these genes. We first tested the expressions of *OsABI1* and *OsABI2*, which are the two genes that interact with ABA receptors and negatively regulate the ABA response. While ABA treatment significantly increased their transcript abundance, *OsKEAP1* downregulation seemed to have little effect on their transcription responses to ABA (Figure 4A,B).

Like *OsABI1* and *OsABI2*, the expression of *OsSnRK2* was induced by the ABA treatment, but its response was not affected by the *OsKEAP1* downregulation (Figure 4C). OsABI3 is known to be upstream of OsABI5 in the ABA signal transduction, though the mechanism is yet unknown. The response of *OsABI3* to ABA seemed similar to that of *OsSnRK2*, but the effect of *OsKEAP1* downregulation on its expression was limited and seemed inconsistent between the two mutant lines (Figure 4D).

OsKEG and OsDSG1 are two post-translational regulators of OsABI5; they keep OsABI5 at low levels in the absence of stress. In contrast to OsSnRK2 and the other genes investigated above, they were neither responsive to ABA treatment nor affected by the *OsKEAP1* downregulation (Figure 4E,F).

### 2.4. Downregulation of OsKEAP1 Altered the Transcription of ABA Response Genes

**Figure 5:**
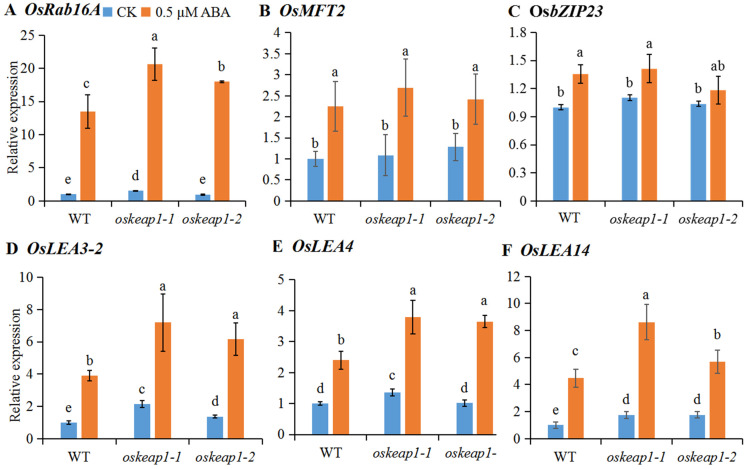
Relative expressions of the ABA response genes and their regulators in germinating seeds that had been germinating for 4 days on a 0.5 MS medium without abscisic acid (ABA) (CK) or with 0.5 μM ABA. The expression levels of the same gene in different materials were compared with the wild-type (WT) germinated without ABA (its level was set as 1). *OsACTIN* was used as an internal reference. Data are shown as the mean ± standard error of three biological repeats. One-way ANOVA was performed for the statistical analysis, where different letters represent significant differences (*p* ≤ 0.05).

OsRab16A is one of the marker ABA response genes with the ABRE in their promoters; it was one of the first few cloned rice genes [30]. OsbZIP23 acts as a transcription factor and OsMFT2 can enhance the binding activity of OsbZIP23 to the *OsRab16A* promoter to activate its transcription [31]. As expected, the transcription of *OsRab16A* was strongly induced by ABA in germinating seeds, with an up-to-13.5-fold increase in the WT (Figure 5A). Its expression in *oskeap1-1* (but not in *oskeap1-2*) was significantly greater than in the WT, even without the ABA treatment. After the ABA treatment, the *OsRab16A* transcription levels in both *oskeap1-1* and *oskeap1-2* were significantly greater than the WT (Figure 5A). The transcriptional response of *OsMFT2* and *OsbZIP23* to ABA seemed far milder than *OsRab16A*, and so was the effect of *OsKEAP1* downregulation (Figure 5B,C). 

Late embryogenesis abundant (LEA) proteins are another type of ABA response gene that is regulated by OsABI5 [16]. We investigated the expression of three representative LEA genes, i.e., *OsLEA3-2* [32], *OsLEA4* [33], and *OsLEA14* [34]. Similar to *OsABI5*, without ABA treatment, all three genes had significantly greater expression in *oskeap1-1*, and *OsLEA3-2* and *OsLEA14* in *oskeap1-2*, as compared with the WT (Figure 5D–F). When germinating with ABA, the expression of all three genes became significantly greater in both *oskeap1* mutants than in the WT.

The ANOVA analysis revealed that there was a significant interaction between the genotype and ABA treatment regarding the transcriptional responses of *OsRab16A* and three *LEA* genes. Eventually, all four of these genes of the WT became significantly less responsive to ABA than those of both *oskeap1* mutants. Among them, only *OsRab16* and *OsLEA14* were shown to be significantly more responsive to ABA in *oskeap1-1* than in *oskeap1-2*.

## 3. Discussion

Our previous study identified and characterized a probable KEAP1 ortholog in rice, i.e., OsKEAP1, and by examination of two downregulated mutants (*oskeap1-1* and *oskeap1-2*) that were generated by genome editing, we demonstrated that its downregulation had a broad range of impacts on plant growth and development, salinity tolerance, ROS homeostasis, and ABA susceptibility [14]. The present study further disclosed that OsKEAP1 interacted with OsABI5 and its downregulation increased the transcription of the *OsABI5* and ABA response genes, which nicely explained the broad phenotype changes of *oskeap1-1* and *oskeap1-2* because OsABI5 (and its orthologs in other plant species) is well known for playing critical roles not only in ABA regulation but also in other biological processes in plant growth and development [15]. In addition, through a comparison of the state-of-the-art working mechanisms of the KEAP1–NRF2 module in animals and the OsKEAP1–OsABI5 module in rice (elaborated below), we predicted that OsKEAP1 is likely to form a complex with OsABI5 and OsKEG and functions in the same manner as KEAP1 does in the KEAP1–NRF2¬–Cul3–E3 ubiquitin ligase complex. Therefore, we argue that, despite the very low homology of KEAP1/NRF2 with their plant counterparts, the OsKEAP1–OsABI5 module in rice (and its orthologs in *Arabidopsis* and other plants) did resemble the KEAP1–NRF2 module in animals in several aspects.

### 3.1. The Likely OsKEAP1–OsABI5 Working Mechanism

In plants, ABA is the best-known stress signaling molecule, though its role as a growth signal is also essential in plant development and senescence. Over the past few decades, the core components of the ABA signaling pathway have been largely identified and characterized, though new genes are still being added (see reviews by Nonogaki [16], Hewage et al. [35], and Sun et al. [36]). Because OsKEAP1 and its orthologs are not part of the ABA signaling pathway in any of the working models proposed so far, no attempt has been made to link the KEAP1–NRF2 module to the ABA signaling module. However, with the identification of OsKEAP1 and its role in OsABI5 regulation, the working mechanism of OsKEAP1–OsABI5 seems to be similar to that of KEAP1–NRF2 to some extent.

Under physiological conditions in animals, NRF2 forms a complex with KEAP1 and Cul3–E3 ubiquitin ligase with KEAP1 as an adaptor, where NRF2 is ubiquitinated and subsequently degraded in the proteasome pathway. Similarly, in plants when there is no or little ABA, ABI5 also forms a complex with a RING-type E3 ligase (KEG) [17] and is S-nitrosylated [19] and subsequently ubiquitinated and destabilized by the 26S proteasome pathway [16,19].

Under oxidative conditions, NRF2 is dissociated from KEAP1 by ROS in a dose-dependent manner [37]. After being released from KEAP1, NRF2 is phosphorylated by various kinases, such as protein kinase C (PKC), mitogen-activated protein kinases (MAPKs), PKR-like endoplasmic reticulum kinase (PERK), phosphatidylinositol 3-kinase (PI3K), and glycogen synthase kinase-3 (GSK-3), the function of the phosphorylation of NRF2 is often kinase-dependent, e.g., PERK enhances the nuclear accumulation of NRF2 under endoplasmic reticulum stress [38], while GSK-3 catalyzes the phosphorylation of NRF2 and decreases its stability independent of Keap1-mediated degradation [39]. Phosphorylated NRF2 is translocated to the nucleus, where it activates the transcription of target genes (including NRF2 itself) with ARE in their promoter [4,5,28].

In the ABA signaling pathway, ABA stabilizes and activates ABI5 via a series of actions: (1) promoting auto-ubiquitination and degradation of KEG, possibly through autophosphorylation of KEG, and thus, ABI5 is free from KEG-mediated degradation, but the actual mechanism leading to the release of ABI5 remains elusive [16]; (2) ABI5 is phosphorylated by SnRK2, which is freed from the PP2C–SnRK2 complex. With the demonstration of OsKEAP1 interacting with OsABI5 in the present study, we might have identified the switch that turns ABI5 to and from KEG, that is, OsKEAP1 forms a complex with OsABI5 and KEG under physiological conditions, while it dissociates OsABI5 from KEG under stressed conditions, which would nicely explain how ABI5 is released from the complex with KEG. Phosphorylated ABI5 then binds to the ABRE motif in the promoter of target genes and activates their transcription [15]. In addition to SnRK2, several other kinases also are reported to mediate ABI5 phosphorylation, such as Ca^2+^ signaling-related kinases, namely, CPK11 [40] and PKS5, which is a member of the CIPK/PKSs [41].

Although ABI5 was first identified as a gene involved in the ABA response [42,43], it is now well known that ABI5 plays a wide range of roles in both plant development and adaptation to abiotic stresses [15]. Abiotic stress often results in the accumulation of both ROS and ABA in plant cells; hence, it is possible that ABI5 is involved in both the ABA and antioxidant responses. Bi et al. [44] demonstrated that ABI5 directly binds to the promoter of *CATALASE 1* (*CAT1*) and activates its expression, and hence showed that ABI5 plays a role in regulating ROS homeostasis in seed germination. In our previous study, we demonstrated that the expressions of two rice catalase genes, namely, *OsCATA* and *OsCATB*, were changed in the *oskeap1* mutants [14], which could now be explained by the increased expression of *OsABI5* in the two mutants.

In summary, based on the elaborations above, we argued that in plants, the OsKEAP1 homologs were indeed bound to ABI5 and formed a complex with KEG under normal physiological conditions, similar to the KEAP1–NRF2–Cul3–E3 complex in animals. Both NRF2 and ABI5 are ubiquitinated and degraded through the proteasome pathway under physiological environments. Under unfavorable environments, NRF2 (under an oxidative environment) and ABI5 (with elevated levels of ABA or under oxidative stress) are released from the complex due to change in the KEAP1 configuration and auto-ubiquitination of KEG; they are then phosphorylated and become active for activating target genes.

**Figure 6:**
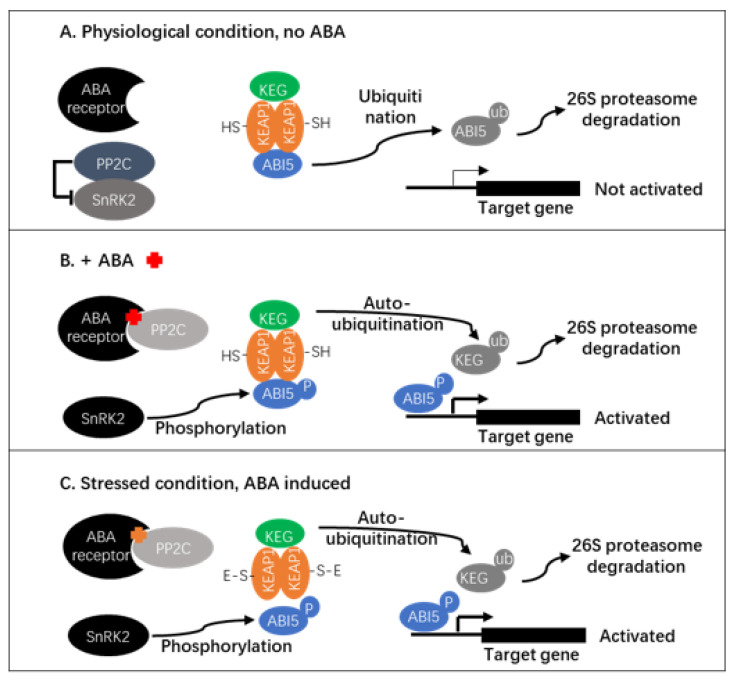
A hypothetical module of KEAP1–ABI5 in plants. Under physiological conditions, ABI5 is degraded and unable to activate its target genes (including itself) (**A**). When there is a high level of ABA (**B**) or under stress (**C**), the configuration of KEAP1 is changed due to reactions with electrophiles, and ABI5 is released from the complex. ABI5 is then phosphorylated and translocated to the nucleus and activates the transcription of target genes.

Because orthologs of OsKEAP1 and OsABI5 are conserved across plant species [14,15], we argue that the KEAP1–ABI5 module is highly likely to act in similar ways in plants. A simplified hypothetical working module of KEAP1–ABI5 in plants is hence proposed in Figure 6 for future studies.

### 3.2. The Effect of OsKEAP1 Downregulation on OsABI5 Expression and Its Consequences

The *oskeap1-1* and *oskeap1-2* mutants were generated via targeted mutagenesis of the 5′ untranslated region (5′UTR) of *OsKEAP1*. Although the transcription of *OsKEAP1* was significantly downregulated in both mutants as compared with that of the WT (Figure 3A) (also in Liu et al. [14]), the abundance of *OsABI5* transcripts was increased significantly only in *oskeap1-1* under normal conditions in both germinating seeds (Figure 3B) and untreated seedlings (Figure 3C). However, when germinating with ABA supplementation or growing under salt stress, the *OSABI5* transcription levels were significantly increased in both mutants as compared with those of the WT (Figure 3B,C). The actual mechanism leading to this discrepancy might be complicated due to the complex and yet not fully understood regulatory process from *OsKEAP1* mRNA to *OsABI5* mRNA. Here are some speculations: under physiological conditions, (1) the different *OsKEAP1* mRNA levels between *oskeap1-2* and the WT might not lead to significantly different OsKEAP1 protein levels; (2) the OsKEAP1 level in *oskeap1-2* was significantly lower than that in the WT, but the amount in *oskeap1-2* was sufficient to sequester as much OsABI5 as in the WT because OsKEAP1 in WT is very likely to be more than sufficient for its function; (3) OsABI5 released from OsKEAP1 needs to be phosphorylated and translocated into the nucleus, and hence, the amount of active OsABI5 that ultimately lands in the nucleus is not necessarily completely proportional to the OsABI5 released from the complex. It is indeed the nuclear OsABI5 that self-activates *OsABI5* transcription via binding to its promoter [15] and ultimately determines the mRNA level of *OsABI5*. Therefore, it should not be surprising to note that the *OsABI5* mRNA levels in *oskeap1-2* and the WT were not significantly different. 

With the presence of ABA in the culture medium, the effect of *OsKAEP1* downregulation on the *OsABI5* expression was amplified due to the effect of both OsKEAP1 and phosphorylation. Salt stress is known to increase ABA production [33], as well as ROS accumulation (as proven in our previous study [14]); hence, although the increase of the *OsKEAP1* mRNA level was less than twofold [14], the *OsABI5* mRNA level was increased to more than 15 times that of both the untreated WT and the *oskeap1* mutants 3 h post treatment, with the two mutants having significantly greater levels than the WT (Figure 3C). 

The effect of *OsKEAP1* downregulation was extended to genes regulated by OsABI5, as shown by the representative ABA response genes (Figure 5A,D–F). Although ABA did induce the transcriptions of *OsABI1*, *OsABI2*, *OsABI3*, and *OsSnRK2* in both the *oskeap1* mutants and the WT (Figure 4A–D), the downregulation of *OsKEAP1* had little effect on their transcription response to ABA. Similarly, the downregulation of *OsKEAP1* did not affect the transcription of *OsKEG* and *OsDSG1* (Figure 4E,F). These results suggest that OsKEAP1 played a role in ABA signaling but not in the regulation of these genes, which is consistent with the role of OsKEAP1 we proposed above. 

### 3.3. Further Studies 

Based on our research results and a comprehensive analysis of the known components and working models of the ABA signaling pathway, we proposed that OsKEAP1 (and its orthologs in plants) should be added to this important pathway as an important regulator of OsABI5 at the post-translational level. We also indicated that the OsKEAP1–OsABI5 module resembles the KEAP1–NRF2 module in certain features, with a potential role in the regulation of the antioxidant response. However, more studies are needed to clear some ambiguities and further elaborate on this module and its functioning mechanisms.

In our previous study, we demonstrated that OsKEAP1 is present in both the cytoplasm and nucleus [14]. In the present study, we localized the OsABI5 protein to the nucleus (Figure S2), which is consistent with Zou et al. [20]. Our BiFC assay using protoplasts demonstrated that OsKAEP1 and OsABI5 were colocalized in the nucleus (Figure 2). Recently, Bhagat et al. [45] also demonstrated, via an in planta BiFC assay, that AtABI5 is located in the nucleus, where it is phosphorylated by AtMPK3. In animals, KEAP1 is mainly located in the cytoplasm with only a small amount in the nucleus and endoplasmic reticulum [29], and phosphorylated NRF2 is translocated to the nucleus and activates its target genes. These observations suggest the different subcellular localizations of the KEAP1/NRF2 and OsKEAP1/OsABI5 complexes. Because the transient transformation of protoplasts and the subsequent preparation for microscopy observation are expected to cause certain levels of damage, which may trigger ROS accumulation, the subcellular localization of OsKEAP1/OsABI5 observed in the present study might not necessarily reflect their actual localization in normal living cells. More studies are needed to corroborate these findings. 

The ABA signaling pathway has been extensively studied in the past few decades, where various components have been identified from the PYRABACTIN RESISTANCE1 (PYR1)/PYR1-LIKE ABA receptors, to their interacting PP2C proteins (ABI1 and ABI2), to proteins that contribute to stabilizing, degrading, and activating ABI5 (such as KEG and SnRK2) [46], to ABI5 interacting proteins (such as OsMFT2 [24] and the CROWED NUCLEI proteins [47]. The present study suggests that OsKEAP1, a probable KEAP1 homolog, is a repressor of ABI5, which is the key gene in the ABA signaling pathway. Due to its importance in ABA signaling and probably also in ROS regulation, it is worthwhile to perform more studies on OsKEAP1 and its orthologs in other plant species, for example, the determination of various functional domains and amino acids that are key to the interaction with OsABI5, which, in turn, will help to elucidate its working mechanism.

The KEAP1–NRF2–ARE module has been considered as a central node for the cross-talk of cellular defense and survival pathways, where the transcription factor NRF2 can transactivate the expression of over 1000 protective genes [48]. Therefore, the KEAP1–NRF2 pathway has become one of the hottest topics in biological research during the past decade, with more than 1500 new papers published each year since 2016 [3]. If further studies confirm that OsKEAP1–OsABI5 is actually a homologous module of KEAP1–NRF2 in rice (and similarly in other plants), we contemplate that it may play roles in plants that are even broader in scope than in animals because it may respond to both ABA and ROS. Therefore, more in-depth studies on this new module will generate valuable insights into plant molecular biology, which will help to improve and/or manipulate plant responses to various abiotic stresses.

## 4. Materials and Methods

### 4.1. Search of NRF2 Orthologs

The amino acid sequence of human NRF2, downloaded from NCBI (https://www.ncbi.nlm.nih.gov/, accessed on 15 February 2018), was used as a query for a BLAST search in the NCBI database to search for the NRF2 orthologs in rice. Multiple databases were further searched to identify the *NRF2* orthologous genes in rice, such as Gramene (http://www.gramene.org/, accessed on 6 May 2018) and RAPDB (https://rapdb.dna.affrc.go.jp/index.html, accessed on 6 May 2018). The protein sequence alignment of NRF2 and OsABI5 was analyzed using MEGA7 software (MEGA, version: MEGA7, Mega Limited, Auckland, New Zealand, USA).

### 4.2. Plant Materials

The *oskeap1* mutants, i.e., *oskeap1-1* and *oskeap1-2*, were developed from a *japonica* rice variety Xidao #1 (WT) by our group using CRISPR/Cas9-mediated target mutagenesis of the untranslated region of *OsKEAP1*, where their characteristics were reported in Liu et al. [14]. Of particular relevance to the present study, it was demonstrated that *oskeap1-1* and *oskeap1-2* had significantly downregulated *OsKEAP1* transcriptions. As compared with their wildtype parent Xidao #1, mutant seedlings became more susceptible to salt and H_2_O_2_ and the germination of mutant seeds was more susceptible to ABA [14].

In the present study, for the investigation of the role and mechanism of OsKEAP1 in the ABA response during seed germination, mature grains of the WT and the two *oskeap1* mutants were dehulled and normal-looking brown rice grains were selected for testing (i.e., those with black spots and wrinkles were not used). They were surface-sterilized via rinsing with 75% ethanol for 30–60 s, followed by soaking in a 1% NaClO solution for 40 min. After rinsing in water 4–5 times, the sterilized grains were cultured on 0.5 MS medium [49] supplemented with 0 and 0.5 μM ABA. They were put in a growth chamber for germination for 4 days and used for the total RNA extraction.

### 4.3. Yeast Two-Hybrids Assay

For the Y2H assay, the coding sequences of *OsKEAP1* and its conserved Kelch repeat domain (*OsKelch*, amino acids 418–654) and *OsABI5* were amplified from rice cDNA with specific primers (Table 1), where the former two were cloned into pGADT7 and the latter was cloned into pGBKT7 (Clontech, Shanghai). The recombinant pGADT7 constructs, i.e., pGADT7–OsKEAP1 and pGADT7–OsKelch, were co-transformed with pGBKT7–OsABI5 into the yeast strain AH109 using the Yeastmaker Yeast Transformation System 2 according to the manufacturer’s instructions (Clontech, Shanghai) [50]. The transformed yeasts were incubated on double dropout (DDO, SD/-Leu/-Trp) plates to test the transformation success. Colonies were collected from DDO plates, incubated with shaking in a liquid DDO medium for 24 h at 30 °C, and then incubated on quadruple dropout (QDO, SD/-Ade/-His/-Leu/-Trp) plates. The growth of transformed yeast cells on QDO plates would indicate strong interactions between the test proteins in 3–5 days.

### 4.4. Subcellular Localization and BiFC Assay

The cDNA of *OsABI5* was PCR amplified using the forward primer OsKeap1GFP-F and reverse primer OsKeap1GFP-R, containing EcoRI and BamHI recognition sites, respectively. The PCR amplicon was cloned into the linearized pTZM28–GFP vector (digested with EcoRI and BamHI) to produce a fusion gene with a green fluorescence protein (GFP) under the control of the cauliflower mosaic virus (CaMV) 35s promoter. The vector was named the *OsABI5::GFP*.

For the subcellular localization of OsABI5, rice protoplasts isolated from 10-day-old rice seedlings were co-transfected with *OsABI5::GFP* and a nucleus marker vector (*NLS-mCherry*) using polyethylene glycol [51] and incubated for 16 h. The pCAMBIA-1301–GFP vector under the control of the CaMV 35S promoter was used as a control. Protoplasts were observed under an LSM780 fluorescence confocal microscope (Carl Zeiss, Oberkochen, Germany) for protein subcellular localization.

For the BiFC assay, the cDNAs encoding the Kelch domain (OsKelch) and OsABI5 were cloned into primary pSAT1 HY105 vectors [52,53]. The generated fusion protein vectors were co-transformed into rice protoplasts using PEG4000 solution [51], with NLS-mCherry used as a nucleus marker. The interactions between OsKelch and OsABI5 were observed in rice protoplasts after being incubated in W5 solution in darkness for 12–16 h and examined using an LSM780 inverted confocal microscope with an Argon laser (Carl Zeiss, Oberkochen, Germany) [54].

The primers used for the construction of vectors used for the Y2H assay, protein subcellular localization, and BiFC analysis are listed in Table 1. All primers were synthesized at Tsingke Biotechnology Co., Ltd. (Hangzhou, China).

### 4.5. Real-Time Quantitative PCR

Total RNA was extracted from germinating seeds that had been germinating for 4 days on 0.5 MS medium without or with 0.5 μM ABA using an RNAprep pure Plant Kit (Tiangen, Beijing, China). They were reverse-transcribed using a PrimerScript RT reagent kit with a gDNA eraser (Takara, Dalian, China). A Hieff^TM^ qPCR SYBR^®^ Master Mix (Yeasen, Shanghai, China) was used for the real-time quantitative PCR (qPCR) analysis. qPCR was performed on a LightCycler 480 (Roche, Penzberg, Germany). The primers used for the analysis of the different genes are listed in Table 1. Three biological replicates and three technical repeats were taken for each treatment and the relative expression levels were calculated using the 2^−ΔΔ*CT*^ analysis method [54], with the rice *Actin* gene as an internal reference.

For the analysis of the *OsABI5* transcription in the 10-day-old rice seedlings subjected to the salt treatment, the cDNAs produced in our previous study [14] were directly used in the present study.

The primers used for the qPCR analysis are listed in Table 2.

### 4.6. Data Analysis

One-way ANOVA was performed for the gene expression data using IBM SPSS Statistics Subscription (SPSS Statistics, Version: Chinese Version of IBM SPSS Statistics Subscription, IBM, Austin, TX, USA); a significant difference was set at *p* ≤ 0.05.

## 5. Conclusions

In this study, we identified OsABI5 as an NRF2 homolog with the highest amino acid identity score in rice. We demonstrated that OsKEAP1 interacted with OsABI5 in the nucleus via its Kelch domain in protoplasts, and the downregulation of *OsKEAP1* could significantly upregulate *OsABI5* and its representative target genes. The effect of *OsKEAP1* downregulation on the expression of *OsABI5* and its target genes became more significant in germinating seeds when ABA was added to the medium, while no effect was observed on the expression of genes that regulated OsABI5 at the post-translational level. In-depth analyses suggested that OsKEAP1 was likely functioning in a way similar to KEAP1 by forming a multiprotein complex in which OsABI5 was ubiquitinated and subsequently degraded under physiological conditions.

## Figures and Tables

**Table 1:** Primers used for the Y2H, BiFC, and subcellular localization analysis.

Name of Primers	Forward Strand (5’–3’)	Reverse Strand (5’–3’)
Y2H analysis		
KEAP1-AD	gccatggaggccagtgaattcATGGGTGCTGGAAAGAAGACTCA	cagctcgagctcgatggatccAATGGCAACGGCGCATGC
Kelch-AD	gccatggaggccagtgaattcGCCCGTGCATATGCGGCT	cagctcgagctcgatggatccGCTGAGTGGGCTCCCCCT
OsABI5-BD	gaattcATGAACATGGACGAGTTTGT	ggatccCCACATGCAGCTGCCGCTGC
BiFC analysis		
Kelch-nEYFP	ctcaagcttcgaattcGCCCGTGCATATGCGGCT	gtcgactgcagaattcGCTGAGTGGGCTCCCCCT
cEYFP-OsABI5	gaattctgcagtcgacATGGCATCGGAGATGAGCA	cgcggtaccgtcgacCCACATGCAGCTGCCGCTGC
Subcellular localization analysis		
OsKEAP1GFP	gaattcATGGGTGCTGGAAAGAAGACTCA	ggatccCAATGGCAACGGCGCATGC
OsABI5GFP	gaattcATGGCATCGGAGATGAGCA	ggatccCCCACATGCAGCTGCCGCTGC

Letters in lowercase represent the sequences in vectors and the letters in uppercase represents the sequences in genes.

**Table 2:** Primers used for the qPCR analysis.

Name of Primers	Gene Numbers	Forward Strand (5’–3’)	Reverse Strand (5’–3’)
qrt-OsKEAP1	*Os01g0165200*	CAAGCACTGGCCAGCTTAAT	GATTAGCGCGAACAGGAGCA
qrt-OsABI5	*Os01g0859300*	GCTAACGACCGACAGGTAACACT	CCATCCCGTTGTACCCACC
qrt-OsABI1	*Os05g0572700*	ACGAGTTGGAACGAGTGGAAGC	GGCTTCAGGTAGTAGTCGCCTATG
qrt-OsABI2	*Os01g0513100*	CGCGATCAATAGGTGACAGA	CGCTAATGTTGTCCTTGCTC
qrt-OsABI3	*Os08g0101000*	CCCAACAACAAAAGCAGGAT	CCTTTGTATTGGACGAGACG
qrt-OsSnRK2	*Os03g0764800*	TGGCAAGACTGCTGATGTATGG	TCGAAAGGATATGCACCAACTACC
qrt-OsKEG	*Os05g0392000*	GGCCAACATGTGCAGCTCAAAC	CTGACCACGCCATCCAAATCTAGG
qrt-OsDSG1	*Os09g0434200*	CCGCTTTGGAAGAATCTCTG	TTCCTGTCTTCCTCCTCTTC
qrt-OsMFT2	*Os01g0111600*	ACGGTGGGGATACACAGGTA	TGTGTTGAAGTTGGGCCTGT
qrt-Rab16A	*Os11g0454300*	CAGCTCAAGCTCGTCTGA	GCTTCTCCTTGATCTTCTCCTT
qrt-OsbZIP23	*Os02g0766700*	AGAGCAATGTGTTCCCTCCG	ATCTTGCCGAAGCCATTGGA
qrt-OsLEA3-2	*Os03g0322900*	AAGATGTGATCCCCCATGAGC	TTCAGCACCACTGCACTTAGA
qrt-OsLEA14	*Os01g0705200*	GATGTACACGCTCGGGATGT	TTCCAGGCTTGTAGGTGCTG
qrt-OsLEA4	*Os01g0705200*	AGTTCATGTAGAGTTCACTTCGCT	GCATCTCCCACAACATGATACC
Actin	*Os03g0836000*	CTTCATAGGAATGGAAGCTGCGGGTA	CGACCACCTTGATCTTCATGCTGCTA

## Supplementary Materials

The following are available online at https://www.mdpi.com/2223-7747/10/3/527/s1. Figure S1: Diagram of human NRF2 and its rice homolog OsABI5 (**A**) and the alignment of their bZIP domain (**B**). aa: amino acid; bZIP: basic region/leucine zipper motif. The bZIP domains were predicted using the ScanProsite database (https://prosite.expasy.org/scanprosite/ accessed on 6 May 2018), Figure S2: Localization of OsKEAP1 and OsABI5 in a rice protoplast. Protoplasts transfected with control vector (35S::GFP) had a bright GFP signal distributed throughout the cell, whereas those with 35S:OsKEAP1::GFP and 35S:OsABI5::GFP had a fluorescent signal (in green) localized in the nucleus and cytoplasm, as confirmed by the nuclear localization sequence (NLS) signal in the nucleus (in red). Scale bar: 5 µm. The same results for 35S:OsABI5::GFP 35S::GFP that were previously reported in Liu et al. [14] are used here for comparison with 35S:OsABI5::GFP, Datasheet 1: Search results for NRF2 homologs in rice (updated on 28 Feb 2021; proteins are listed with “percentage of identity” as the main priority).
