# Supplementary material for: OsKEAP1 Interacts with OsABI5 and Its Downregulation Increases the Transcription of OsABI5 and the ABA Response Genes in Germinating Rice Seeds
Source: Plants (Basel). 2021 Mar 11;10(3):527. doi: 10.3390/plants10030527 (PMC8001349; doi:10.3390/plants10030527)
Supplement: Supplementary file 1 [file plants-10-00527-s001.zip › Supplementary_Figures 1 and 2.docx]

Article

OsKEAP1 Interacts with OsABI5 in Nucleus and Its Downregulation Affects Transcription of ABA Signaling and Response Genes in Germinating Rice Seeds

Yan-Hua Liu^1^, Meng Jiang^1^, Rui-Qing Li^2^, Jian-Zhong Huang^1,3^ and Qing-Yao Shu^1,^*

^1^ National Key Laboratory of Rice Biology and Zhejiang Provincial Key Laboratory of Crop Germplasm Resources, Institute of Crop Sciences, Zhejiang University, Hangzhou 310058, China; 11616004@zju.edu.cn (Y.-H.L.); mengjiang@zju.edu.cn (M.J.); 11416095@zju.edu.cn (R.B.); jzhuang@zju.edu.cn (J.-Z.H.)

^2^ College of Agronomy, Anhui Agricultural University, Hefei 230036, China; 2018065@ahau.edu.cn

^3^ Key Laboratory for Nuclear Agricultural Sciences of Zhejiang Province and Ministry of Agriculture and Rural Affairs, Institute of Nuclear Agricultural Sciences, Zhejiang University, Zijingang Campus, Hangzhou 310058, China

***** Correspondence: qyshu@zju.edu.cn

| 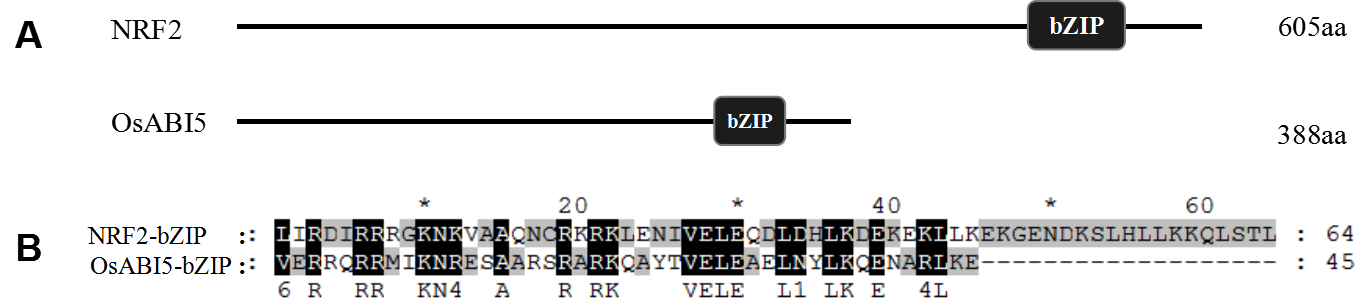 |
| --- |
| **Figure S1.** Diagram of Human NRF2 and its rice homolog OsABI5 (**A**) and alignment of their bZIP domain (**B**). aa: amino acid; bZIP: basic region/leucine zipper motif. The bZIP domains were predicted using the ScanProsite database ( https://prosite.expasy.org/scanprosite/). |

| 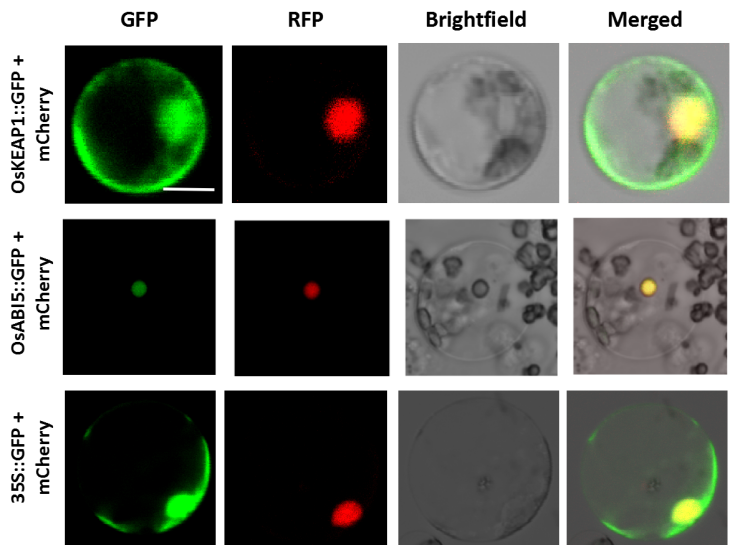  **Figure S2.** Localization of OsKEAP1 and OsABI5 in rice protoplast. Protoplasts transfected with control vector (35S::GFP) have a bright GFP signal distributed throughout the cell, whereas those with 35S:OsKEAP1::GFP and 35S:OsABI5::GFP have a fluorescent signal (in green) localized in the nucleus and cytoplasm, confirmed by nuclear localization sequence (NLS) signal in the nucleus (in red). Bar = 5 µm. Same results of 35S:OsABI5::GFP 35S::GFP previously reported in Liu et al. (2020) are used here for comparison with 35S:OsABI5::GFP. |
| --- |
